# A giant *Pseudomonas* phage from Poland

**DOI:** 10.1007/s00705-013-1844-y

**Published:** 2013-09-26

**Authors:** Zuzanna Drulis-Kawa, Tomasz Olszak, Katarzyna Danis, Grazyna Majkowska-Skrobek, Hans-W. Ackermann

**Affiliations:** 1 Institute of Genetics and Microbiology, University of Wroclaw, Przybyszewskiego 63/77, 51-148 Wroclaw, Poland; 2 Department of Microbiology-Infectiology and Immunology, Medical School, Laval University, Quebec, 1050, avenue de la Médecine, Quebec, G1X 4C6 Canada

**Keywords:** Bacteriophage, DNA size, Giant, *Myoviridae*, *Pseudomonas*

## Abstract

A novel giant phage of the family *Myoviridae* is described. *Pseudomonas* phage PA5oct was isolated from a sewage sample from an irrigated field near Wroclaw, Poland. The virion morphology indicates that PA5oct differs from known giant phages. The phage has a head of about 131 nm in diameter and a tail of 136 × 19 nm. Phage PA5oct contains a genome of approximately 375 kbp and differs in size from any tailed phages known. PA5oct was further characterized by determination of its latent period and burst size and its sensitivity to heating, chloroform, and pH.

## Introduction

Nearly 6,000 tailed bacteriophages have be examined in the electron microscope, making them the largest viral group known. They contain double-stranded DNA and constitute the order *Caudovirales*, which is divided into three families: the *Myoviridae*, with long contractile tails (about 25 % of observations, e.g., coliphage T4), the *Siphoviridae*, with long noncontractile tails (57 %, represented by coliphage λ), and the *Podoviridae*, with short tails (14 %, exemplified by coliphage T7) [1]. All three groups vary enormously in size, DNA content, physicochemical properties, and structural elements such as collars, tail fibers and spikes. Clearly, tailed phages are highly evolved and enormously diversified. Myovirus bacteriophages, particularly of enterobacteria, *Bacillus*, and *Pseudomonas* tend to be larger than sipho- and podoviruses. Inordinately large phages with DNA contents of over 200 kbp have been called “jumbo bacteriophages” [2, 3]. They have been found in bacteria as diverse as *E. coli,* vibrios, *Sphingomonas* [2], pseudomonads [2, 4], *Ralstonia* [3, 5], and *Yersinia* [6, 7]. A few, found in *Aeromonas* and *Vibrio*, with DNA contents of 233-244 kbp, have typical T4 morphology and slightly elongated heads [8]. These “jumbo phages” consist of a heterogeneous mixture of medium-sized phages and truly giant viruses that dwarf anything in the viral world except the recently discovered mimiviruses of the protozoon, *Acanthamoeba* [9] and some phycodnaviruses [10]. The very largest bacteriophage known, *Bacillus megaterium* phage G, has a head of 160 nm in diameter and a tail of 455 nm in length (Table 1). We present here a *Pseudomonas aeruginosa* myovirus that is larger than any *Pseudomonas* phages known, is the third-largest bacterial virus after *Bacillus* phage G, and possibly represents a novel genus of bacterial viruses.

**Table 1:** A gallery of the largest phages in the literature

Host	Phage	Dimensions [nm]		DNA [kbp]	Calibration	References
		Head	Tail length			
*Alicyclobacillus acidoterrestris*	P10	152	240		catalase	[18]
*Bacillus megaterium*	G	160	453	497	catalase	[2, 8, 26] GenBank JN638751.1
*Caulobacter crescentus*	φCr26	160	140		diffraction grating replica	[24]
	φCr24	140	140			
*Cronobacter sakazakii*	GAP32	115	118	358	T4 tails	Abbasifar (conference report)
*Gluconobacter oxydans*	GW6210	170	136	~275	TMV	[25]
*Escherichia coli*	121Q	116	115	341	catalase	[2, 23]
	PBECO4	132	125	348	none	[19]
*Klebsiella sp.*	RaK2	123	128	346	catalase, T4 tails	[20, 21]
*Proteus vulgaris*	121	150	150		none	[22]
*Pseudomonas aeruginosa*	PA5oct	131	136	375	T4 tails	this work
*Ralstonia solancearum*	φRSL1	123-150	105-138	231	λ phage	[3, 5]
Unknown	X	156	119		catalase	[16]

kbp, kilobase pair; nm, nanometer; TMV, tobacco mosaic virus

## Materials and methods

### Phage isolation

The *Pseudomonas aeruginosa* PAO1 (ATCC 15692) strain, purchased from the American Type Culture Collection (ATCC), was used as a host for phage propagation. A water sample taken from irrigated fields was centrifuged at 15,000 g for 15 min, and the supernatant was filtered through a 0.22-μm Millex-GP filter (Merck Millipore, Darmstadt, Germany, SLGP033RS). One ml of filtered water sample and 0.5 ml of a broth culture of *P. aeruginosa* PAO1 strain, grown overnight in TSB, were added to 10 ml of TSB and incubated at 37 °C for 18 h. The suspension was then centrifuged again and filtered through a 0.22-μm Millex-GP filter. This procedure was repeated three times to eliminate any bactericidal activity of chemicals possibly present in the water samples. The presence of bacteriophage and the titre in the filtrate were determined by the appearance of plaques on the bacterial lawn using the double-agar layer technique [11]. Phage PA5oct was deposited in the Félix d’Herelle collection at Laval University under the accession number HER 483.

### Electron microscopy

A filtered high-titer phage lysate was centrifuged at 25,000 × g for 60 min, using an Beckman (Palo Alto, CA) J2-21 centrifuge and a JA19.1 fixed-angle rotor. The pellet was washed twice in ammonium acetate (0.1 M, pH 7.0) under the same conditions. Phages were deposited on copper grids with carbon-coated Formvar films and stained for 10 s with uranyl acetate (2 %, pH 4.5) or phosphotungstate (2 %, pH 7). Excess liquid was blotted off and phages were examined under a Philips EM 300 electron microscope. The magnification was calibrated using T4 phage tails as a standard [12].

### Pulsed-field gel electrophoresis (PFGE) analysis

A 10-ml sample of filtered phage lysate with a titer of 10^8^ PFU/ml was concentrated by centrifugation (15,000 g for 25 min) using a Sigma 3-30 K Spincontrol Comfort centrifuge with a 19776 rotor (Sigma Zentrifugen GmbH, Germany) in a solution of 1 M NaCl and 10 % PEG 6,000 (Acros Organics, Geel, Belgium, 192280010). The sedimented precipitate was suspended in 1 ml of TE buffer (1 mM EDTA, 10 mM Tris, pH 8.0). Sample plugs were prepared by mixing equal volumes of concentrated phage suspension with melted and cooled (40 °C) low-melting-point agarose (BioShop® Canada Inc., Burlington, ON, AGA101). Prepared blocks were placed in lysis buffer (50 mM Tris, 50 mM EDTA, 1 % SDS) and digested for 2 h with proteinase K at 54 °C. After digestion, plugs were rinsed four times with TE buffer. DNA samples were separated on a 1 % agarose gel using a Bio-Rad (Hercules, CA, U.S.A.) CHEF-DR III system (16.5 h, 6 V/cm, 12 °C, switch time 1-50, angle 120°). Yeast Chromosome PFG Marker (New England Biolabs, Ipswich, MA, U.S.A., N0345S) was used as a size marker.

### One-step growth

One-step growth was determined according to the method of Pajunen *et al.* [13], with modifications. An equal volume of a mid-exponential bacterial culture in TSB was mixed with phage suspension (2.5×10^6^ PFU/ml) to obtain a multiplicity of infection of 0.01. Phages were allowed to adsorb for 8 min at 37 °C, after which time the mixture was diluted to 10^−4^. Triplicate samples were taken at 5-min intervals for titration.

### Sensitivity of PA5oct to heat, chloroform, and pH

The thermal resistance of PA5oct at various temperatures (40, 50, 60, 70, and 80  °C) was determined by incubating the phage (9.7×10^8^ PFU/ml) at the respective temperature for 5, 15, 30, 45 and 60 min at pH 7.4 in TSB. Bacteriophage titers were assessed by the double agar layer technique [11]. Chloroform sensitivity was estimated by incubating equal volumes of phage suspension (1.9×10^9^ PFU/ml) and chloroform for 1 h at room temperature and 4 °C with intermittent shaking, followed by titration. The pH sensitivity was studied by inoculating a volume of 100μl of bacteriophage suspension (9.7×10^9^ PFU/ml) into 900 μl of TSB medium of pH 3-11. Phages were titrated after incubation for 1 h and 24 h at room temperature.

## Results

**Fig. 1:**
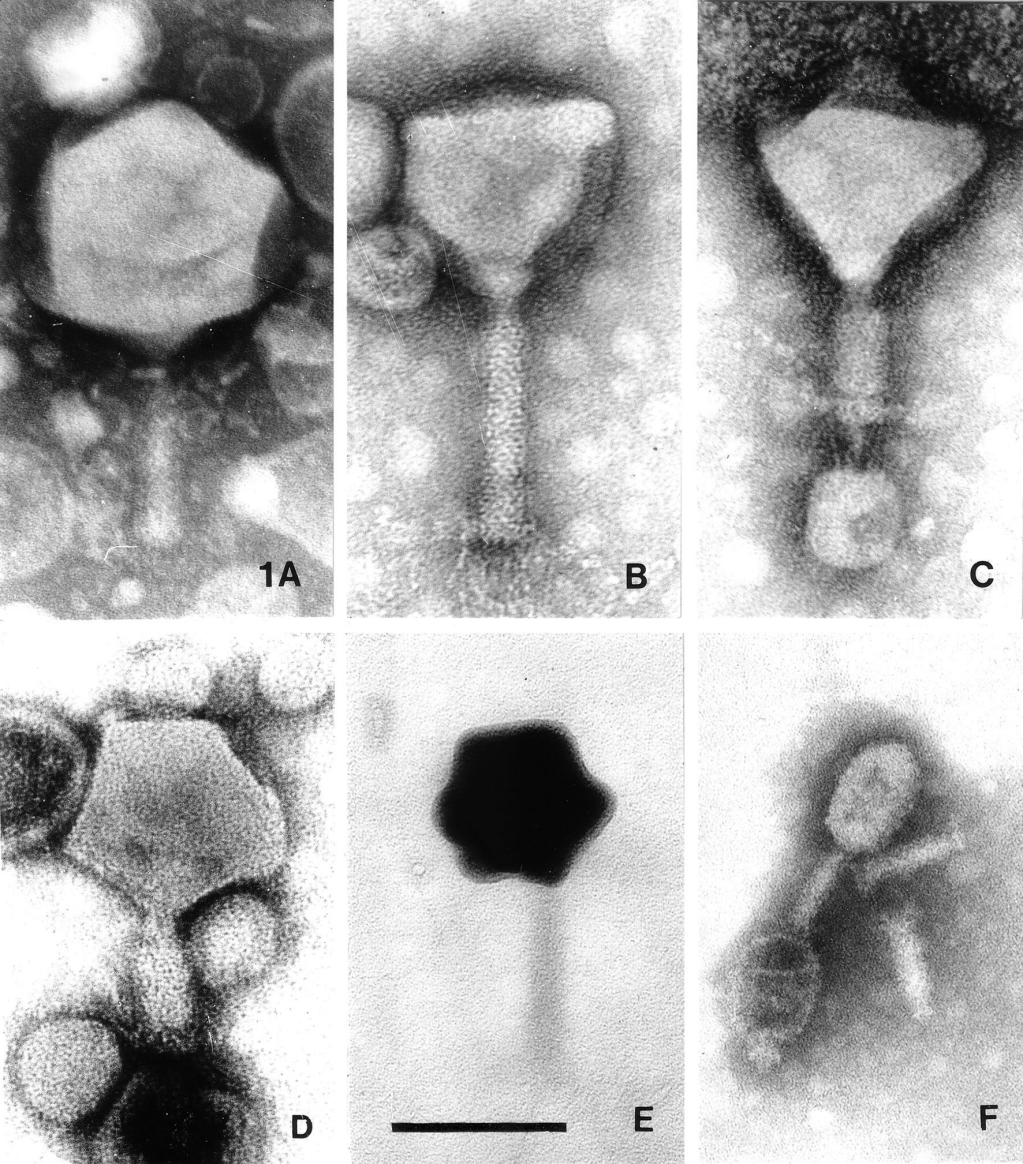
Phage PA5oct and a giant and a very small myovirus for comparison. **A** the X particle with extended tail. Note its enormous head and relatively short tail [16]. **B** to **E** phage PA5oct. **B** normal phage with an extended tail; **C** chance observation of a phage with a contracted tail and base plate, adsorbed to small bacterial debris (the triangular aspect of the phage head is normal); **D** phage with a pentagonal head and a contracted tail; **E** phage with a positively stained head (such heads are always shrunken and should not be measured); **F** *Bdellovibrio bacteriovorus* phage φ1402. The picture shows one intact phage, two isolated tails, and an empty head [17]. **A** and **F** phosphotungstate; **B** to **E**, uranyl acetate. Final magnification, 297,000×; the bar indicates 100 nm

**Fig. 2:**
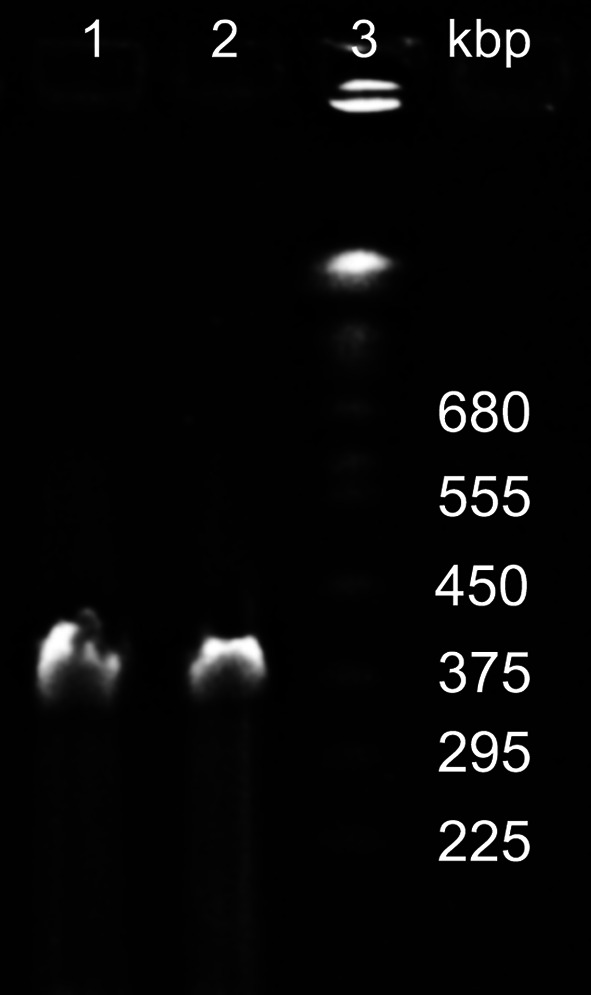
PFGE analysis of PA5oct phage DNA (lanes 1 and 2); Yeast Chromosome PFG Marker (lane 3)

Phage PA5oct, alternatively named vB_PaeM_PA5oct in a new nomenclature system [14], was isolated from sewage collected from irrigated fields in a wastewater treatment plant near Wroclaw, Poland. Phage titers were 10^8^-10^9^ PFU/ml with 1-mm-wide clear plaques on soft agar (0.7 %). Electron microscopy showed extremely large, mostly intact myoviruses and very large amounts of cell debris (Fig. 1). Heads measured 131 nm between opposite apices (60 measurements) and were icosahedral as shown by the simultaneous observation of capsids with hexagonal and pentagonal outlines. Unlike *Pseudomonas* phage φKZ [15], no inclusion bodies were observed in empty phage heads. Tails measured 136 × 19 nm in the extended state and consisted of a neck of 10 nm, a sheath with about 25 transverse striations, and an indistinct base plate of 33 × 7-10 nm with short fibers of 30 × 2 nm. No collar was observed, but many phages carried amorphous material at the neck. Contracted tail sheaths measured 50 × 30 nm and were separated from the base plate by a space of about 2 nm. As determined by PFGE, the genome size of PA5oct was about 375 kbp (Fig. 2). The genome of PA5oct is going to be sequenced.

One-step growth experiments indicated a latent period of 40 min and a burst size of about 30-40 phage particles per infected bacterial cell. Phage PA5oct was stable at a temperature of 40-60 °C, where no reduction of PFU/ml was observed over a period of 60 min. More than 60 % of phages remained infective after a 15-min incubation at 70 °C, while only 13% phages were alive after a 60-min incubation at this temperature. By contrast, after 5 min of incubation at 80 °C, 98 % of phages lost their activity. No reduction in infectivity was observed after treatment with chloroform at a concentration of 50 % for 1 h at room temperature or at 4 °C. Phage particles were relatively stable within a pH range of 5-11 after incubation at room temperature (almost 90 % of phages were alive), with the exception of 24 h of incubation at pH 11.0, where a 50 % reduction in phage activity was observed. When exposed to pH 3.0 for 60 min, phage titers dropped by 10 % and after 24 h of incubation, <20 % of phages were still alive.

## Discussion

PA5oct is larger than the largest *Pseudomonas* phages known, namely the φKZ phage group of *P. aeruginosa*, whose members are characterized by head diameters of about 124 nm and a tail length of 184 nm [4, 15]. It is also larger than phage Lu11 of *P. putida*, which has nearly the same dimensions, but has three or more curly fibers attached to its tail [4]. Phage PA5oct is truly unique among *Pseudomonas* phages, of which about 550 have been studied by electron microscopy [1].

The largest myovirus known, and also the largest phage in terms of capsid size and DNA content, is *Bacillus megaterium* phage G. It has a head of 160 nm in diameter and contains a DNA of 497,513 bp [GenBank accession number JN638751.1]. The next-largest phages with seemingly secure dimensions are phages P10 of *Alicyclobacillus acidoterrestris*, PA5oct, GAP32 of *Cronobacter sakazakii*, coliphage 121Q, and an entity called the X particle that was observed in the fluid of crushed silkworm larvae and never propagated [16]. Fig. 1 shows PA5oct, the X particle [16], and *Bdellovibrio bacteriovorus* phage φ1402, which is the smallest known autonomous myovirus and which has a genome of only 23,931 bp [17]. P10 was obtained from Germany for inclusion in the Félix d’Herelle collection at Laval University (www.phage.ulaval.ca) and was photographed [18], but it was lost for unknown reasons. Phage GAP32 is a member of an emerging group of large phages, which possibly represent a new genus [Abbasifar R, Kropinski AM, Sabour PM, Ackermann H-W, Lavigne R, Alanis Villa A, Nash JHE, Vandersteegen K, Griffiths MW. 2013. Supersize me: *Cronobacter sakazakii* phage GAP32. Proc Viruses of Microbes, Brussels, Belgium, July 16-20, 2012]. It comprises coliphage 121Q, phage PBECO4 of *E. coli* 157:H6, and perhaps *Klebsiella* phage RaK2 [19–21]. The members of this group are characterized by very large heads, relatively short tails, and limited genomic relationships with other phages. Most of these viruses were measured after calibration of magnification with catalase crystals (Table 1).

Although genome size determination and genome sequencing are now common techniques, some giant phages, especially those reported in the 1970s or 1980s, were identified by electron microscopy only. The literature includes a series of phages with uncertain or dubious dimensions, obtained without or with questionable magnification controls. *Proteus* phage 121 was published as early as 1969, apparently without a control [22]. Indeed, H.-W.A. found a morphologically identical coliphage, 121Q, that appeared substantially smaller [23]. The size of *Caulobacter* phages φCr24 and φCr26 was determined by means of diffraction grating replicas, an internal standard that is good for low magnification (x30-40,000 only) and totally unsuitable for high magnification (x200-300,000) [24]. *Gluconobacter* phage GW6210 is certainly a giant virus, but it was measured by calibration with tobacco mosaic virus (TMV). The latter is fragile and disintegrates easily into small rods of variable length. This suggests that the *Gluconobacter* phage was measured after calibration with virus fragments [25]. *Ralstonia* phage φRSL1 was measured after calibration with phage λ which has a flexible tail and appears intrinsically inappropriate as a size standard. The published micrographs of φRSL1 were unsatisfactory, but Yamada *et al*. later obtained excellent images and measured a head diameter of 125 nm, which suggest that φRSL1 is related to the GAP32 group [personal communication, 5].

Size determination is one of the weakest points in viral electron microscopy. Even if the right test specimens are used, calibration of magnification is problematic. The adjustment of the electron microscope or photographic enlarger can be a difficult procedure and, if wrongly done, a source of errors. Beef liver catalase crystals are the only internal standard available for calibration at high magnification, but the crystals are fragile and disintegrate easily. Instead of catalase crystals, H.-W.A. has been using uranyl acetate-stained T4 phage tails for many years. When calibrated with catalase, they are 114 nm long, which equals 33-34 mm at a magnification of x300,000 [12]. T4 tails are stable for years, large enough to be measured with some certainty, and small enough to be numerous in a field. Other standards have also been used, e.g., TMV crystals, but they have not won acceptance. In conclusion, phage PA5oct appears to be the third-largest phage known, after *Bacillus* phage G and *Alicyclobacillus* phage P10.

## Abbreviations

PFU: Plaque-forming unit
g: Unit of gravity
TMV: Tobacco mosaic virus

## References

[1] Ackermann H-W, Prangishvili D (2012). Prokaryote viruses studied by electron microscopy. Arch Virol.

[2] Hendrix RW (2009) Jumbo bacteriophages. In: Van Etten JL, (ed.), Lesser known large dsDNA viruses. Curr Top Microbiol Immunol 328:229–240, Berlin, Springer 19216433

[3] Yamada T, Satoh S, Ishikawa H, Fujiwara A, Takeru Kawasaki T, Fujie M, Ogata H (2009) A jumbo phage infecting the phytopathogen *Ralstonia solanacearum* defines a new lineage of the *Myoviridae* family Virology 398:135–137. doi:10.1016/j.virol.2009.11.043 10.1016/j.virol.2009.11.043 20034649

[4] Krylov VN, Dela Cruz DM, Hertveldt K, Ackermann H-W (2007) ‘‘φKZ-like viruses’’, a proposed new genus of myovirus bacteriophages. Arch Virol. 152:1955–1959. doi:10.1007/s00705-010-0783-0 10.1007/s00705-007-1037-7 17680323

[5] Effantin G, Hamasaki R, Kawasaki T, Bacia M, Moriscot C, Weissenhorn W, Yamada T, Schoehn G (2013). Cryo-electron microscopy three-dimensional structure of the jumbo phage φRSL1 infecting the phytopathogen *Ralstonia solanacearum*. Structure.

[6] Kiljunen S, Hakala K, Pinta E, Huttunen S, Pluta P, Gador A, Lonnberg H, Skurnik M (2005). Yersinophage φR1-37 is a tailed bacteriophage having a 270 kb DNA genome with thymidine replaced by deoxyuridine. Microbiology.

[7] Skurnik M, Hyytiäinen HJ, Happonen LJ, Kiljunen S, Dattta N, Mattinen L, Williamson K, Kristo P, Szeliga M, Kalin-Mänttäri L, Ahola-Iivarinen E, Kalkkinen N, Butcher SJ (2012). Characterization of the genome, proteome, and structure of yersiniophage φR1-37. J Virol.

[8] Lavigne R, Darius P, Summer EJ, Seto D, Mahadevan P, Nilsson AS, Ackermann H-W, Kropinski AM (2009) Classification of *Myoviridae* bacteriophages using protein sequence similarity. BMC Microbiol 9: 224 doi:10.1186/1471-2180-9-224 10.1186/1471-2180-9-224 PMC2771037 19857251

[9] La Scola B, Audic S, Robert C, Jungang L, de Lamballerie X, Drancourt M, Birtles R, Claverie JM, Raoult D (2003) A giant virus in amoebae Science 299 (5615) 20233; PMID 12663818; doi:10.1126/science.1081867 10.1126/science.1081867 12663918

[10] Dunigan DD, Fitzgerald LA, Van Etten JL (2006). Phycodnaviruses: a peek at genetic diversity. Virus Res.

[11] Adams MH (1959). Bacteriophages.

[12] Ackermann HW (2009) Basic phage electron microscopy. *In* Bacteriophages: Methods and Protocols, Vol. 1, eds. A.M. Kropinski and M. Clokie. Ser. Methods in Molecular Biology, 501, 113–126. Humana Press, Totowa, NJ 10.1007/978-1-60327-164-6_12 19066816

[13] Pajunen M, Kiljunen S, Skurnik M (2000). Bacteriophage φYeO3-12, specific for *Yersinia enterocolitica* serotype O:3, is related to coliphages T3 and T7. J Bacteriol.

[14] Kropinski A, Prangishvili D, Lavigne R (2009). Position paper: the creation of a rational scheme for the nomenclature of viruses of Bacteria and Archaea. Environ Microbiol.

[15] Krylov VN, Smirnova TA, Minenkova IB, Plotnikova TG, Zhazikov IZh, Khrenova EA (1984). *Pseudomonas* bacteriophage φKZ contains an inner body in its capsid. Can J Microbiol.

[16] Ackermann H-W, Auclair P, Basavarajappa S, Emadi Konjin HP, Savanurmath C (1994). Bacteriophages from *Bombyx mori*. Arch Virol.

[17] Ackermann H-W, Krisch HM, Comeau AM (2011). Morphology and genome sequence of phage φ1402: A dwarf myovirus of the predatory bacterium *Bdellovibrio bacteriovorus*. Bacteriophage.

[18] Ackermann H-W, Azizbekyan RR, Emadi Konjin HP, Lecadet M-M, Seldin L, Yu MX (1994) New *Bacillus* bacteriophage species Arch Virol 135:333–344 10.1007/BF01310018 7979971

[19] Kim MS, Hong SS, Park KS, Myung H (2013) Genomic analysis of bacteriophage PBECO4 infecting *Escherichia coli* 0157:H7 Arch Virol. pii:1007/SC00705-03-1718-3 10.1007/s00705-013-1718-3 23680925

[20] Simoliunas E, Kaliniene L, Truncaite L, Klausa V, Zajanckauskaite A, Meskys R (2012) Genome of *Klebsiella* sp.-infecting bacteriophage vB_KleM_RaK1 J Virol 86:5406. doi:10.1128/JVI.00347-12 10.1128/JVI.00347-12 PMC3347331 22492928

[21] Simoliunas E, Kalienine L, Truncaite L, Zajanckauskaite A, Staniulis J, Meskys AR. (2013) *Klebsiella* phage vB_KleM-RaK2–a giant singleton virus of the family *Myoviridae* PLoS One 8(4) e60717. doi:10.1371/journal.pone.0060717 10.1371/journal.pone.0060717 PMC3622015 23593293

[22] Nacesco N, Constantinesco SP, Petrovici A (1969). Aspects électrono-optiques du phage convertisseur *Proteus vulgaris* 121. Arch Roum Pathol Exp Microbiol.

[23] Ackermann H-W, Nguyen T-M (1983). Sewage coliphages investigated by electron microscopy. Appl Environ Microbiol.

[24] Johnson RC, Wood NB, Ely B (1977). Isolation and characterization of bacteriophages for *Caulobacter crescentus*. J Gen Virol.

[25] Robakis NK, Palleroni NJ, Despreaux CW, Boublik M, Baker CA, Churn PJ, Claus GW (1985). Isolation and characterization of two phages for *Gluconobacter oxydans*. J Gen Microbiol.

[26] Ageno M, Donelli G, Guglielmi F (1973). Structure and physico-chemical properties of bacteriophage G II. The shape and symmetry of the capsid. Micron.

